# Mechanism, kinetics and microbiology of inhibition caused by long-chain fatty acids in anaerobic digestion of algal biomass

**DOI:** 10.1186/s13068-015-0322-z

**Published:** 2015-09-15

**Authors:** Jingwei Ma, Quan-Bao Zhao, Lieve L. M. Laurens, Eric E. Jarvis, Nick J. Nagle, Shulin Chen, Craig S. Frear

**Affiliations:** Department of Biological Systems Engineering, Washington State University, Pullman, WA USA; National Renewable Energy Laboratory, Golden, CO USA

**Keywords:** Algae, Anaerobic digestion, LCFA inhibition, Calcium, Kinetic model, Microbial community

## Abstract

**Background:**

Oleaginous microalgae contain a high level of lipids, which can be extracted and converted to biofuel. The lipid-extracted residue can then be further utilized through anaerobic digestion to produce biogas. However, long-chain fatty acids (LCFAs) have been identified as the main inhibitory factor on microbial activity of anaerobic consortium. In this study, the mechanism of LCFA inhibition on anaerobic digestion of whole and lipid-extracted algal biomass was investigated with a range of calcium concentrations against various inoculum to substrate ratios as a means to alleviate the LCFA inhibition.

**Results:**

Whole algal biomass of *Nannochloropsissalina* represents high lipid content algal biomass while lipid-extracted residue represents its low lipid counterpart. The anaerobic digestion experiments were conducted in a series of serum bottles at 35 °C for 20 days. A kinetic model, considering LCFA inhibition on hydrolysis, acidogenesis as well as methanogenesis steps, was developed from the observed phenomenon of inhibition factors as a function of the LCFA concentration and specific biomass content or calcium concentration. The results showed that inoculum to substrate ratio had a stronger effect on biogas production than calcium, and calcium had no effect on biogas production when inoculum concentration was extremely low. The microbial community analysis by high-throughput Illumina Miseq sequencing indicated that diversity of both bacterial and methanogenic communities decreased with elevation of lipid concentration. Hydrolytic bacteria and aceticlastic methanogens dominated bacterial and archaea communities, respectively, in both high and low LCFA concentration digesters.

**Conclusions:**

This study demonstrated that inoculum concentration has a more significant effect on alleviating LCFA inhibition than calcium concentration, while calcium only played a role when inoculum concentration met a threshold level. The model revealed that each functional microbial group was subject to different levels of LCFA inhibition. Although methanogens were the most susceptible microbes to LCFA inhibition, the inhibition factor for hydrolytic bacteria was more highly affected by inoculum concentration. The microbial community analysis indicated that the bacterial community was affected more than the methanogenic community by high LCFAs concentration. Syntrophic acetogens were sensitive to high LCFA concentrations and thus showed a decreased abundance in such an environment.

**Graphical abstract:**
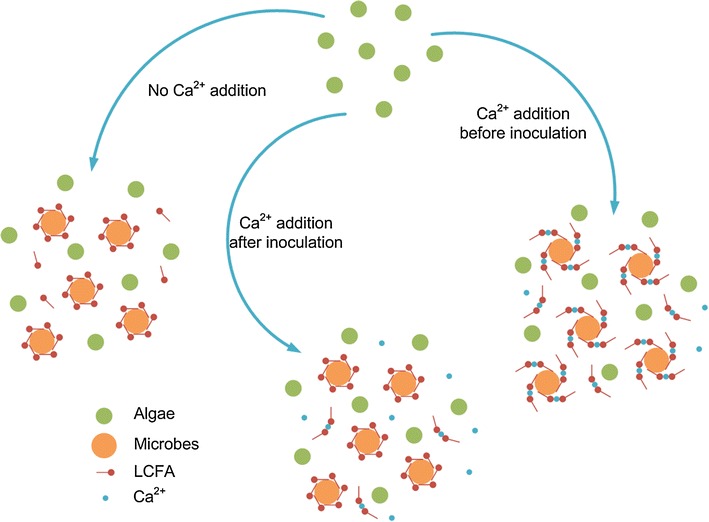
Proposed mechanism of calcium mitigated LCFA inhibition

## Background

Oleaginous microalgae offer a promising option for sustainable production of renewable transportation fuels while reducing lifecycle greenhouse gas emissions relative to fossil fuels [1]. Several studies point to both the expense and inefficiencies of algae lipid-extraction techniques, demonstrating that the importance anaerobic digestion (AD) could have on either whole-algae or residue-algae utilization, respectively, with regard to biofuel/bioenergy production [2, 3].

During the AD process, lipids are initially hydrolyzed to long-chain fatty acids (LCFAs) and glycerol in a fast step by extracellular lipases excreted by hydrolytic bacteria. LCFAs then adsorb to and are transported within microbial cell membranes. Once inside, LCFAs are further degraded to acetic acid and hydrogen through β-oxidation by syntrophic acetogenic bacteria. In lipid-containing substrates, degradation of LCFAs via β-oxidation is the slowest conversion step and controls the overall kinetics of the digestion process [4, 5]. The difference between the rates of hydrolysis of lipids and β-oxidation of LCFAs could result in a reactant–product imbalance and LCFA accumulation over time, resulting in inhibition on microbial activity.

The inhibitory effect of LCFAs on microbial activity of hydrolytic bacteria, acidogens, acetogens, and methanogens within anaerobic consortium has been well documented [6–11]. Methanogens were reported to be more susceptible to LCFA inhibition compared to acidogens [9, 11], while acetotrophic methanogens are reported to be more severely affected than hydrogenotrophic methanogens [8, 10]. If the microbial population is disrupted by LCFAs, inhibited digestion will occur, leading to volatile fatty acids (VFA) accumulation and depressed methane production [12].

Microbial cell membranes, where various essential processes occur, are a primary target of LCFAs. Although the inhibition mechanism of LCFAs on microbial cell membranes is not completely clear, it can be categorized as biochemical and physical in nature. Biochemical inhibition of LCFAs is correlated with its amphipathic structure. Due to detergent properties, LCFAs act as detergent and solubilize the lipid bilayer or membrane proteins, leading to cell lysis [13], enzyme activity inhibition [14], and electron transport chain disruption [15]. The inhibition activity of LCFAs is affected by its structure as well. LCFAs with longer carbon chains tend to be more problematic to microbes than LCFAs with shorter carbon chain [15]. LCFAs with more carbon double bonds can be more problematic than same length LCFAs with saturated carbon bonds [14, 16], and the inhibition effect of LCFAs is positively related to the number of double bonds in the LCFAs [15].

The physical absorption of LCFAs to the surface of microbial cell membranes can lead to mass transfer limitation [7, 17, 18]. Product diffusion and nutrient uptake are affected by LCFA concentration [6] as well as the LCFA:biomass ratio [19], although Rinzema et al. [4] found that the LCFA:biomass ratio is less important than LCFA concentration. Mass transfer limitation could also be a result of LCFAs undermining of transporter proteins located on the membrane or reduction of the proton motive force for active transport [15].

Efforts to reduce the inhibitory effect of LCFAs are needed to maintain an efficient and stable digestion process. Various strategies, including co-digestion [20], addition of adsorbents [8, 21], or discontinuous feeding [22], have been used for overcoming LCFA inhibition. Continuous or pulse exposure of LCFAs has been suggested to acclimate microorganisms for an elevated tolerance to LCFAs [7, 22]. Calcium has been used to reduce the inhibitory effect of LCFAs [8, 23–25], which could be attributed to LCFAs’ precipitation in the form of fatty acid calcium salts [8].

The purpose of this research is to investigate LCFA degradation during AD, focusing in particular on the effect of the calcium:LCFA ratio against the LCFA:biomass ratio. A kinetic model was developed with the consideration of an inhibition factor as a function of LCFA concentration and specific biomass content or calcium ion concentration. Individual inhibition factors for each function group were considered rather than a lumped parameter. Both hydrolytic bacteria and methanogen community structure were characterized by high-throughput sequencing technology Illumina Miseq to evaluate the community structure shift under LCFA inhibition.

## Results and discussion

### Effect of calcium concentration against inoculum to substrate ratio

**Fig. 1:**
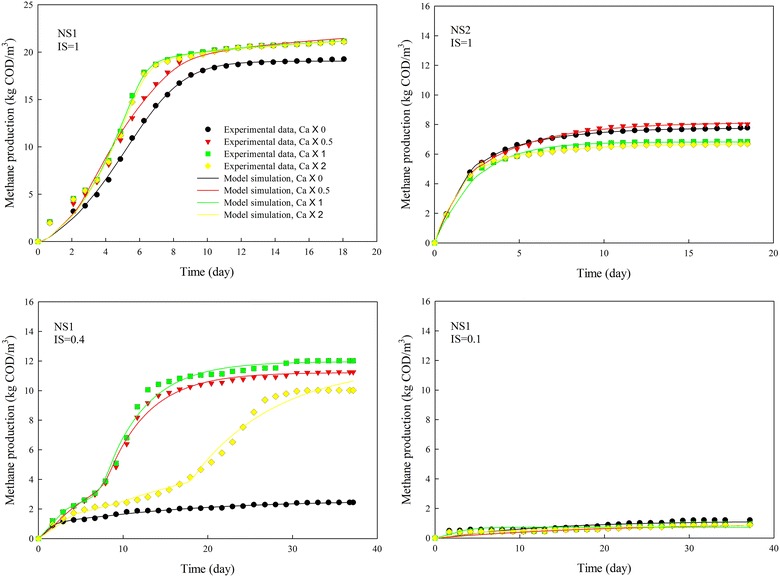
Effect of inoculum to substrate ratio on methane production from anaerobic digestion of NS1 and NS2 with different concentrations of calcium. IS is inoculum to substrate ratio, and Ca is calcium to LCFA ratio

Inoculum to substrate ratio (*I*/*S*) had a significant effect on biogas production (Fig. 1). When *I*/*S* ratio was lower than 1, the AD process was severely inhibited in the high lipid concentration (NS1) digester. The calcium dosed digester showed enhanced biogas production by 10 % as well as accelerated reaction rate at *I*/*S* ratio of 1 in both the NS1 and low lipid concentration (NS2) digesters. At *I*/*S* ratio of 1, calcium dosing with calcium to LCFAs ratio of 0.5 noticeably increased methane production while further increase of calcium to LCFAs ratio had barely any effect on biogas production. Actually, a single calcium ion could bond with two LCFA molecules, so that the calcium to LCFA ratio of 0.5 would be ideal if calcium and LCFAs were completely mixed. Further increase in calcium concentration could not bond more LCFAs, leading to no effect on free LCFA concentration.

**Fig. 2:**
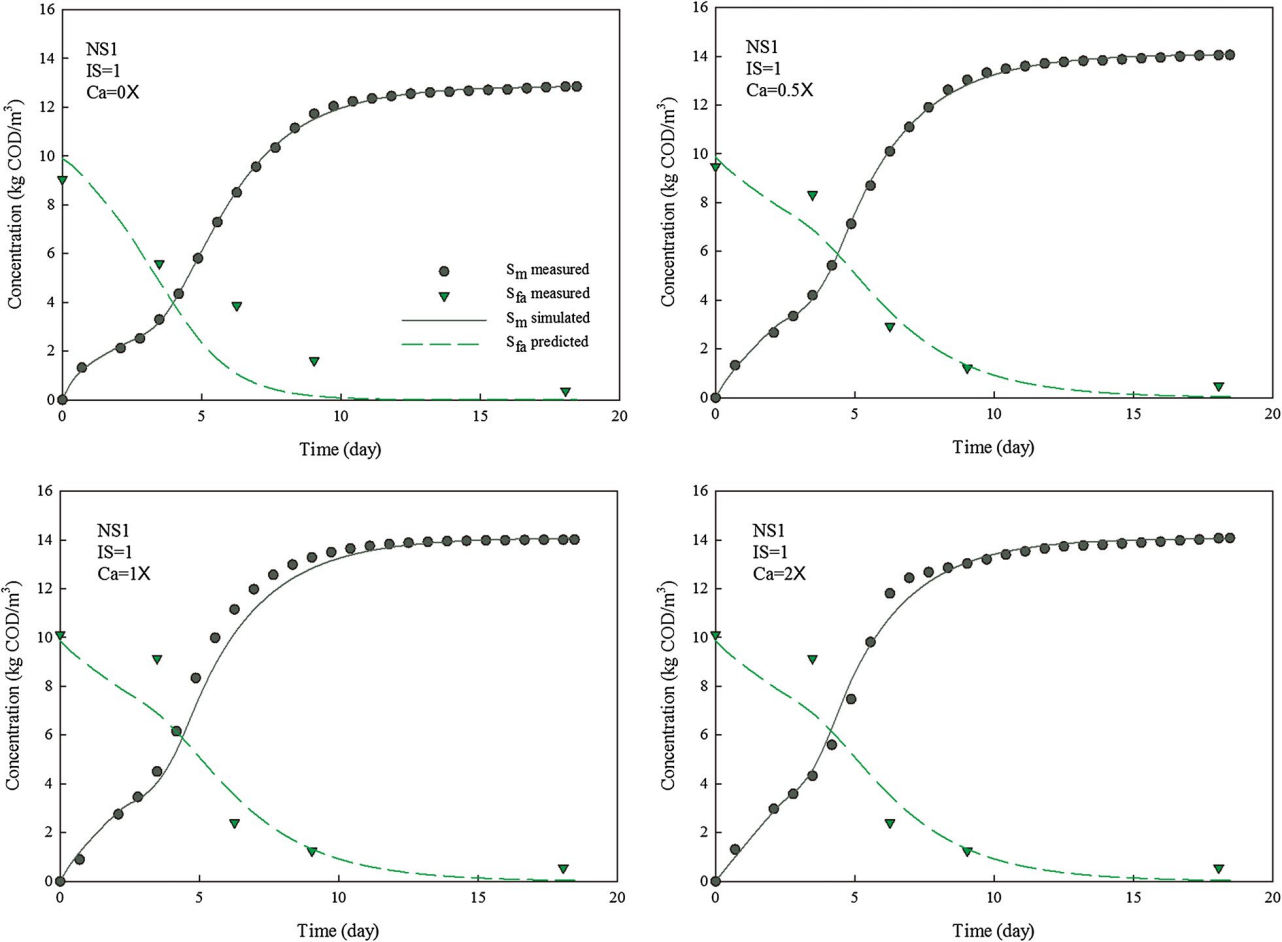
Methane production and LCFA degradation during anaerobic digestion of NS1 with different concentrations of calcium. IS is inoculum to substrate ratio, and Ca is calcium to LCFA ratio

**Fig. 3:**
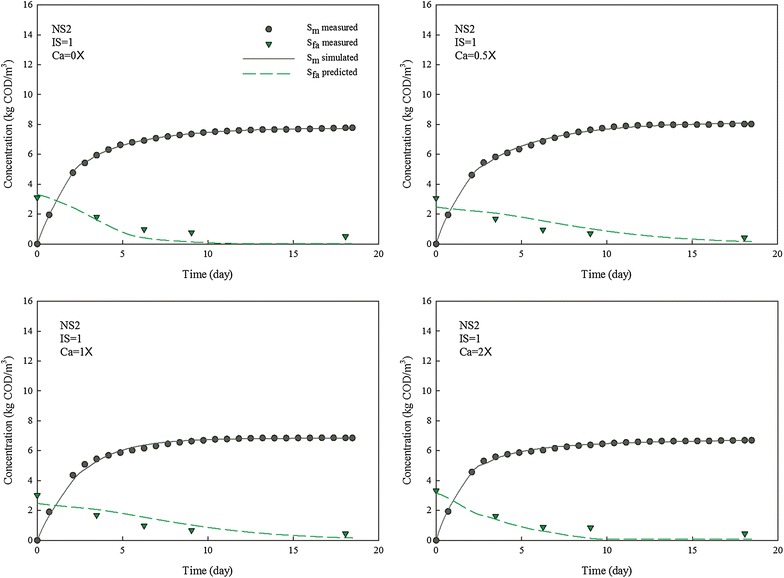
Methane production and LCFA degradation during anaerobic digestion of NS2 with different concentrations of calcium. IS is inoculum to substrate ratio, and Ca is calcium to LCFA ratio

Calcium had no effect on biogas production when the *I*/*S* ratio was extremely low, even with high concentration of calcium. A possible explanation was at such a low inoculum concentration, although calcium was added, the slow methanogenesis step controlled the whole process, which led to VFA accumulation other than LCFAs as the main inhibitor. The same explanation could be applied when the *I*/*S* ratio was 0.4: the released and free algal cells with use of high concentration of calcium raised the hydrolytic rate, but with an unmatched increase in methanogenic rate, the system generated accumulated VFA, prolonging the lag phase and low gas production due to VFA inhibition. The high VFA concentration and low pH were also observed by Zhao et al. [2] when digesting algal biomass at low inoculum concentration. It was noticed that LCFA concentration was higher in digesters with calcium addition compared with control, which led to a delay in the degradation of LCFAs for all digesters with calcium dosage (Figs. 2, 3).

**Fig. 4:**
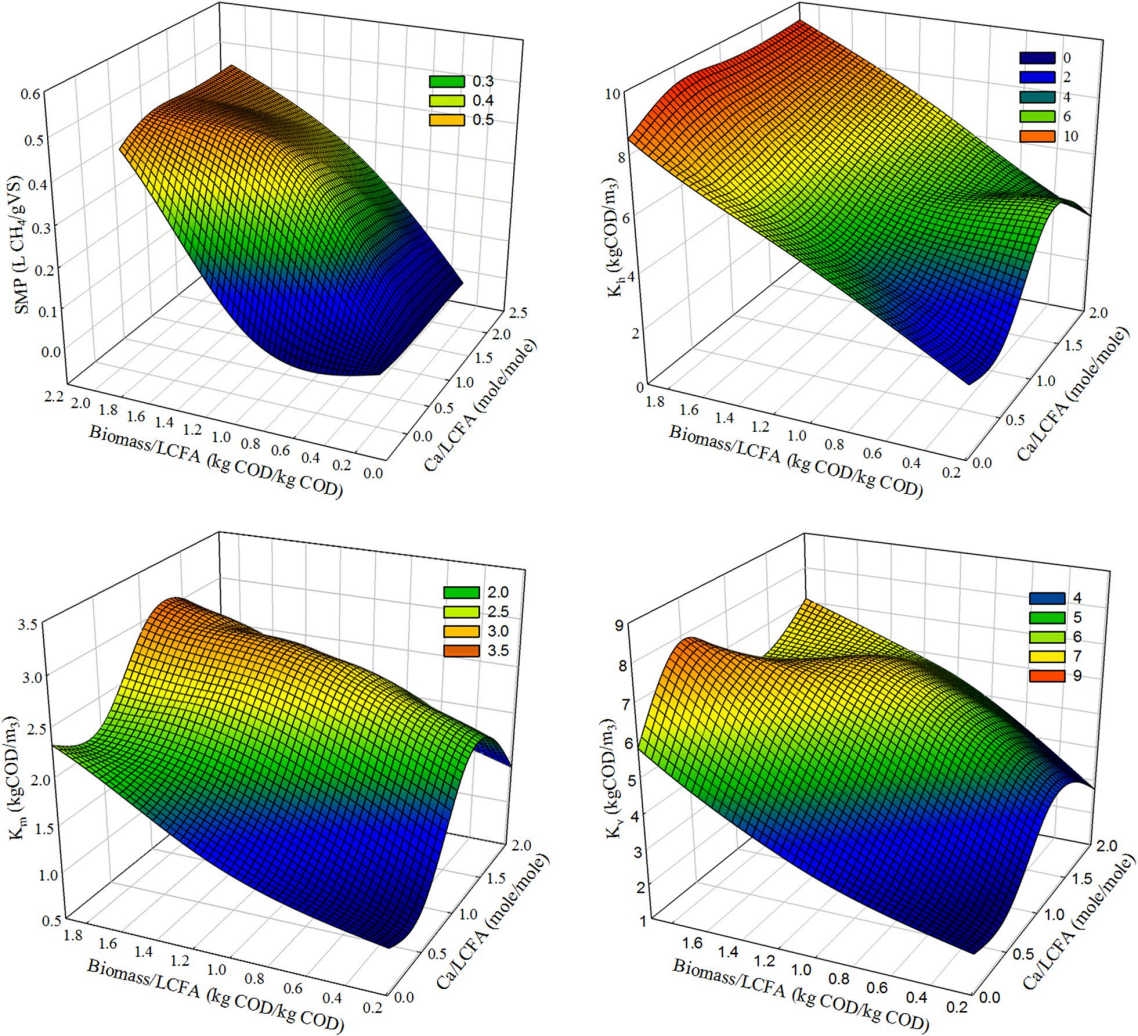
Effect of biomass to LCFA ratio and Ca to LCFA ratio on SMP and inhibition factors

The modeled relationship of specific methane production (SMP) with inoculum:LCFA ratio and calcium:LCFA ratio is illustrated in Fig. 4. It can be seen that inoculum concentration had greater effect on SMP than calcium concentration. Sufficient inoculum was extremely important for healthy digestion without inhibition. With high inoculum:LCFA ratio of 1.0, SMP could reach the value reported by [2] (0.56 and 0.38 L CH_4_/g VS for NS1 and NS2, respectively). Palatsi et al. [21] confirm this observation, detailing that increases in inoculum concentration are the most efficient and fast recovery strategy for an LCFA-inhibited digestion process.

In this research, the LCFA concentration in NS1 and NS2 digesters was 9.9 g COD/L and 3.1 g COD/L, respectively, noticeably higher than the approximate inhibitory threshold range (~0.5–1.5 g COD/L) mentioned in literature [6, 8, 26, 27]. Severe inhibition occurred in digesters with low inoculum concentration, as noticed by extremely low methane production. However, no inhibition was observed for digesters with appropriate *I*/*S* ratio and proper calcium dosing. It seems that high inoculum concentration could be used as a mean of alleviating the inhibition mediated by LCFAs. Calcium ion could also be an effective way to bond LCFAs and thus keep microbial cells from being tightly wrapped by LCFAs. The impact of calcium ion, however, is dependent on the concentration of inoculum, in which a minimum inoculum concentration is required.

### Kinetic analysis of inhibition on anaerobic digestion of algal biomass

The accumulated methane production curves for NS1 and NS2 at various *I*/*S* ratio and calcium concentration were simulated with the developed kinetic model (Figs. 2, 3). The LCFA degradation profiles were then predicted with the developed model.

The inhibition of LCFAs on anaerobic microbial consortia has been kinetically investigated as $K_{\text{I}}$ with a range of 1.3–3.4 kg COD/m^3^ [28, 29]. However, the extent of inhibition varies among hydrolytic bacteria, acidogens and methanogens. Thus, a lumped inhibition factor $K_{\text{I}}$ for whole anaerobic microbial consortium is not sufficient to kinetically describe the different inhibition effect of LCFAs on each microbial group. In this research, the inhibition of LCFAs was evaluated based on individual microbial groups for a more accurate estimation. The results show that inhibition factors for hydrolytic bacteria ($K_{\text{h}}$), acidogenic bacteria ($K_{\text{v}}$), and methanogens ($K_{\text{m}}$) were in the range of 2.6–9.4, 2.1–7.9, and 1.0–2.9 kg COD/m^3^, respectively (Fig. 4). The data suggested a more severe LCFA inhibition on methanogens than on hydrolytic bacteria and acidogens. As a first time kinetic evidence of LCFA inhibition on different functional groups, methanogenesis could be the rate-limiting step in an LCFA-inhibited digestion process, which is consistent with previous research [9, 11].

The *I*/*S* ratio had a remarkable effect on each inhibition factor, with regard to its role in affecting SMP. However, kinetic behavior of each microbial group varies against the change of *I*/*S* ratio. As the *I*/*S* ratio increased from 0.1 to 1.0, $K_{\text{h}}$, $K_{\text{v}}$ and $K_{\text{m}}$ boosted from 2.6, 2.1 and 1.0 to 8.5, 5.8 and 2.3 kg COD/m^3^, respectively, when calcium was not added. Apparently, the inhibition factor of hydrolytic bacteria was most affected by inoculum concentration while that of methanogen was less affected.

Calcium ion concentration showed a limited effect on inhibition factors and the effects on each inhibition factor were similar. However, these effects were dependent on *I*/*S* ratio. The value of inhibition factors doubled with calcium dosing at low *I*/*S* ratio while the impacts of calcium ion concentration were less significant at high *I*/*S* ratio.

This is the first research that kinetically investigated individual inhibition factors for hydrolytic bacteria, acidogens and methanogens, respectively, rather than a lumped inhibition factor for whole microbial consortium by LCFAs. In previous research, one inhibition factor was used for all biological process including hydrolysis, acidogenesis and methanogenesis [17, 19, 29, 30]. The LCFA model developed in this study provided new insights regarding dynamics of the LCFA inhibition process and showed a different inhibition level on each function group. Methanogens were the most fastidious group and were severely impacted by LCFAs; thus, methanogenesis could be the rate-limiting step during AD. Although hydrolytic bacteria were inhibited by LCFAs, and were most impacted by *I*/*S* ratio, hydrolysis could be considered the fastest step. Acidogens were also inhibited by LCFAs, one of its products, which led to acidogenesis being a self-limiting step. However, under the condition without LCFA inhibition, hydrolysis is still the rate-limiting step in anaerobic digestion of microalgae, in which pretreatment could play a role.

### Microbial community structure analysis with Illumina Miseq sequencing

**Fig. 5:**
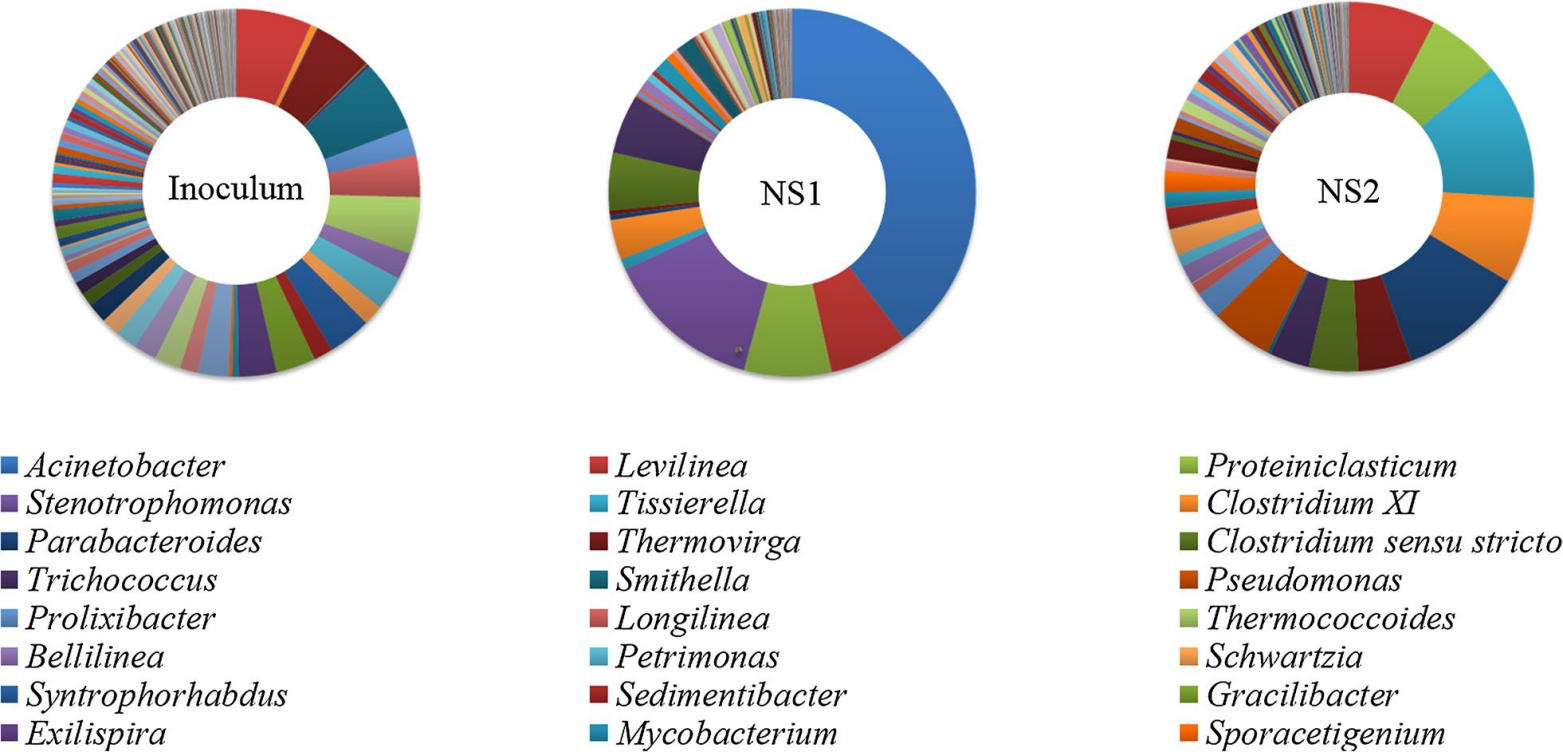
Bacteria communities of inoculum, NS1 and NS2 digester. Relative abundance was defined as the number of sequences affiliated with that taxon divided by the total number of sequences per sample. Legends were only shown for genus making up more than 2 % of total composition

Two samples from digesters fed with NS1 and NS2, respectively, at *I*/*S* ratio of 1 without calcium addition as well as original inoculum were subject to microbial community structure analysis. In total, 36,825 bacteria sequences for 3 samples were classified into 591 genera. The difference of phylum distribution was observed between the two digesters. NS1 digester was dominated by *Proteobacteria*, followed by *Chloroflexi*, and *Firmicutes*, while *Firmicutes*, *Bacteroidetes*, *Chloroflexi*, and *Proteobacteria* were dominant in NS2 digester with balanced abundance. Moreover, *Gammaproteobacteria* belonging to Phylum *Proteobacteria* was enriched in both digesters. The genus level identification of the bacteria communities is illustrated in Fig. 5. Bacteria community in original inoculum showed a balanced population with high diversity. Bacteria community in the NS1 digester showed a distinct pattern with domination of *Acinetobacter* (blue, 39.6 %), *Levilinea* (red, 7.0 %), *Proteiniclasticum* (green, 7.7 %), and *Stenotrophomonas* (purple, 13.9 %). *Acinetobacter* was reported to be the main strain among several pure cultures degrading lipid-containing wastewater with efficient lipase secretion capability [31, 32]. This correlates well with the domination of *Acinetobacter* in the NS1 digester. The bacterial community in the NS2 digester was dominated by *Levilinea* (red, 7.6 %), *Tissierella* (light blue, 11.9 %), *Proteiniclasticum* (green, 6.4 %), *Clostridium* (orange, 7.6 %), and *Parabacteroides* (dark blue, 11.0 %). The population analysis demonstrates that a clearly different microbial community structure was formed in the two digesters due to different lipids loading, although hydrolytic/acidogenic bacteria dominated both NS1 and NS2 digesters.

*Stenotrophomonas* is responsible for the hydrolysis and fermentation of carbohydrate and amino acids [33]. Syntrophic acetogens, including *Clostridium*, *Smithella*, *Tissierella*, *Syntrophorhabdus*, *Sedimentibacter* and *Sporacetigenium,* also presented in the two digesters, although the concentrations were low. Interestingly, the abundance of syntrophic acetogens in the NS1 digester (10.8 %) was significantly lower than that in the NS2 digester (28.2 %), suggesting that syntrophic acetogens were more sensitive to high lipid concentration.

**Fig. 6:**
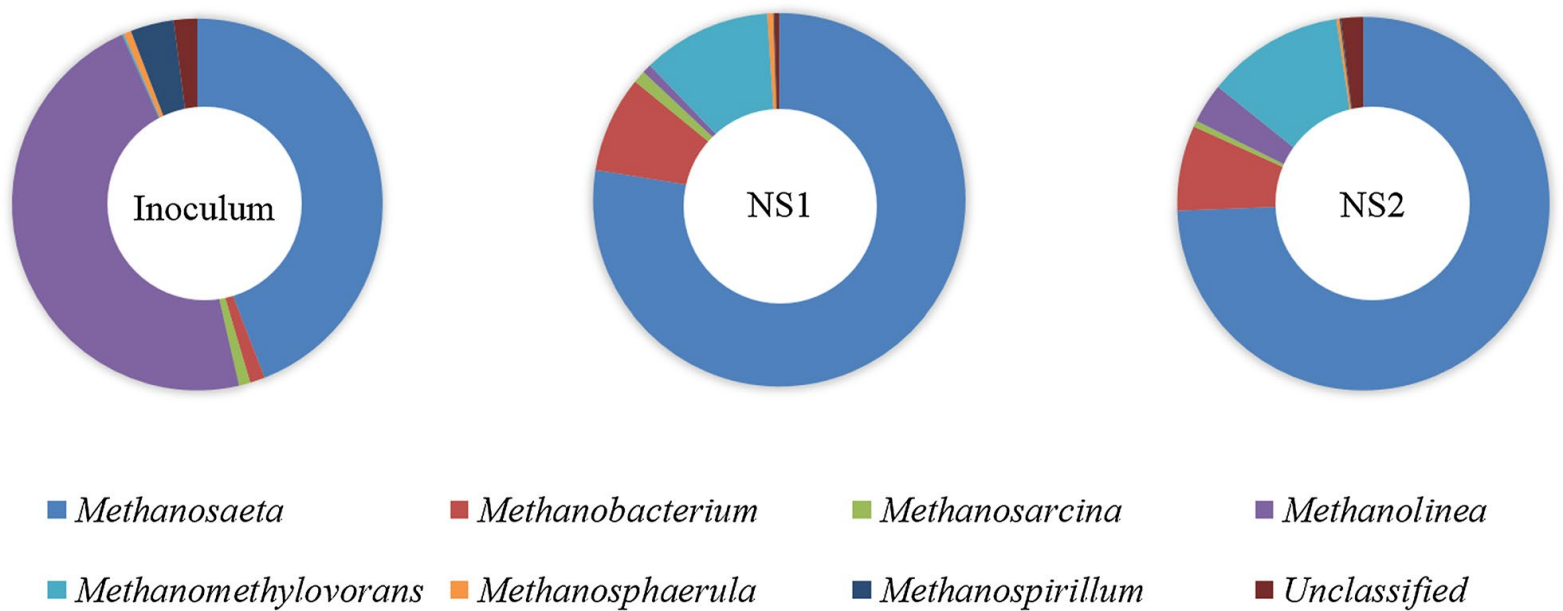
Archaea communities of inoculum, NS1 and NS2 digester. Relative abundance was defined as the number of sequences affiliated with that taxon divided by the total number of sequences per sample

Methanogenic archaea communities were analyzed in the three samples, with a total of 14,220 reads affiliated to 15 genera and 3 orders. The genus level identification of the archaea communities is illustrated in Fig. 6. Methanogenic archaea community in the original inoculum was dominated by *Methanolinea* (purple, 47.0 %), a strict hydrogenotrophic genus, and Methanosaeta (blue, 44.1 %), a strict aceticlastic methanogen genus. However, *Methanosaeta* (blue) prevailed in both of the communities of the NS1 and NS2 digesters (77.6 and 74.4 %, respectively), followed by two hydrogenotrophic genera, *Methanobacterium* (red, around 8 %) and *Methanomethylovorans* (light blue, around 11 %) in both digesters, indicating that aceticlastic methanogenesis was the main pathway for methane formation in the two digesters, regardless of the different lipids content. Dominance of *Methanosaeta* was also found in the anaerobic reactors treating microalgal biomass which was attributed to the low levels of acetate [34].

### Proposed mechanism of calcium mitigated LCFA inhibition

Inhibition of LCFAs could be mainly attributed to physical attachment on the surface of microbial cells. As microbes are coated by LCFAs, limitations on transportation hinder substrate access and subsequent biogas release [17]. Calcium ions could bond free LCFAs, thus reducing the amount of LCFAs available for microbial cells to half of original LCFA concentration (Graphic abstract). This reduction delayed LCFA degradation, compared with the control. Moreover, the steric hindrance effect of calcium bonded LCFAs could further mitigate LCFA inhibition by loosening the LCFA coat. However, calcium ions could not exclusively compete the LCFAs from the surface of microbial cells. As a result, calcium ion addition could not help mitigate LCFA inhibition for those LCFAs already attached on the surface of microbial cells. Moreover, the effect of calcium ion was limited, and it only played a role when the microbial concentration reached a minimal requirement.

It is foreseeable that multivalent ions, ferric ion for example, could bond more LCFAs according to its charge and give rise to a more sophisticated steric hindrance effect, leading to a stronger effect on alleviating LCFA inhibition while using a reduced amount. The optimal multivalent ion to LCFA ratio would be reciprocal to the value of its charge. However, the same rule as calcium still applies, in that it could not relieve LCFAs inhibition after LCFAs attached to the surface of microbial cells.

## Conclusion

High inoculum concentration is the key for a healthy process when digesting high concentration of LCFAs. Inoculum concentration had a more pronounced effect on overcoming the inhibition of LCFAs than that of calcium ion, while calcium ion plays a role only when inoculum concentration met a threshold level. Calcium ion could bond with free LCFAs available to the surface of microbial cells and reduce half of the original LCFAs concentration. Kinetic modeling revealed a remarkable difference among the inhibition factors for each function group of microorganisms. Although methanogens were the most susceptible microbes to LCFA inhibition, the inhibition factor for hydrolytic bacteria was more highly affected by inoculum concentration. The bacterial community was affected more than the methanogenic community by high concentration of LCFAs. Diversity of both bacterial and methanogenic communities decreased with elevation of lipid concentration in the digester. Hydrolytic bacteria and aceticlastic methanogens dominated bacterial and archaea communities, respectively, in both high and low LCFA concentration digesters. Syntrophic acetogens were sensitive to high LCFA concentrations and thus showed a decreased abundance in such environment.

## Methods

### Microalgae and inoculum

*Nannochloropsissalina* (Solix BioSystems) was selected as it was the algal biomass with greatest availability and had lipid content emblematic of industrial strains. Whole algal biomass of *Nannochloropsissalina* (NS1) represents high lipid content algal biomass while lipid-extracted residue (NS2) represents its low lipid content counterpart. Freeze-dried solid biomass of both NS1 and NS2 was provided by Solix BioSystems, Inc. (CO, USA). Lipid in NS1 was extracted with a 3:2 mixture of hexane/isopropanol at 70 °C and 1500 psi [2]. Detailed characteristics of NS1 and NS2 are listed in Table 1. Anaerobic sludge was sampled from an anaerobic digester at the Pullman Wastewater Treatment Facility with TS of 17.1 g/L and VS of 11.7 g/L.

**Table 1:** Chemical composition of *Nannochloropsis* *salina* (NS1) and lipid-extracted residue (NS2)

Composition	NS1	NS2
Algal lipid (%)	37.2	11.8
Carbohydrates (%)	11.5	17.0
Protein (%)	17.2	26.7
Unknown (%)	27.2	34.1
VS/TS (%)	93.0	89.7

### Effect of calcium addition and inoculum to substrate ratio on methane production

A series of biochemical methane potential (BMP) assays were set up to investigate the effect of calcium addition and inoculum to substrate ratio (*I*/*S*) on methane production from NS1 and NS2. The experimental design considered treatments with calcium (CaCl_2_·2H_2_O, Sigma) at concentrations of 0.5, 1 and 2 times that of the algal lipid concentration (mole/mole) against *I*/*S* ratios of 0.1, 0.4 and 1.0 (gVS/gVS). All BMP assays were conducted in serum bottles with working volume of 150 mL and headspace of 100 mL. No additional external nutrients/trace elements were added to the BMP bottles as it was assumed that basic nutrient requirements for anaerobic microorganisms were provided by the wastewater-based inoculum [35]. Algal biomass was added to the serum bottles to impose an organic loading rate of 10 gVS/L, and mixed with CaCl_2_·2H_2_O at designed calcium concentration, before inoculum was added according to the respective *I*/*S* ratio. Before experiments were initiated, each bottle was flushed with N_2_ gas for 15 min to induce anaerobic conditions, and then incubated in a 16-cell automated Challenger AER System (Fayetteville, AR, USA) maintained at 35 ± 1 °C and mixed continuously with a magnetic stirrer set to 200 rpm. Daily methane production was monitored via scrubbing of carbon dioxide with sodium hydroxide pellets containing color indicator.

### Chemical analysis

The analysis for TS, VS and COD was done according to the standard methods [36]. The volume of biogas from the digester was determined by water displacement method. Contents of CH_4_ and CO_2_ were determined via a Varian gas chromatograph (Palo Alto, CA, USA) equipped with a thermal conductivity detector [37].

Lipids were analyzed as fatty acid methyl esters after a one-step acid catalyzed in situ trans-esterification reaction using a GC-FID (Agilent 6890N) equipped with an HP-5 ms capillary column (30 m × 0.25 mm id × 0.25 µm) according to the procedure of Laurens et al. [38]. Protein was calculated from elemental N content [39]. Carbohydrates were determined by H_2_SO_4_ acid hydrolysis followed by HPLC measurement of monosaccharides [40].

### DNA extraction

At the end of the digestion experiment, samples of initial saved inoculum and mixed liquor from the two digesters fed with NS1 and NS2 at *I*/*S* ratio of 1 with no calcium addition were collected. Genomic DNA was extracted and purified using the PowerSoil DNA isolation kit (Mo Bio Laboratories, Inc., CA, USA) according to the manufacturer’s instructions.

### Illumina Miseq sequencing on V4–V5 regions of 16S rRNA genes

The V4–V5 hypervariable region of the 16S rRNA gene was amplified with region-specific primers designed to include Illumina adaptor and barcode sequences (518F-926R for bacteria, 518F-958R for archaea) [41, 42]. Generation of sample amplicons was performed using a double round of PCR and dual indexing on PTC-200 DNA Engine Peltier Thermal Cycler (Bio-Rad Laboratories, Inc., CA, USA). The first round of PCR extracts the targeted regions (here 16S V4 to V5) with initial denaturation at 95 °C for 2 min, 20 cycles of denaturing at 95 °C for 1 min, annealing at 51 °C for 1 min and extension at 68 °C for 1 min, plus a final extension at 68 °C for 10 min. The second round of PCR attaches the sample barcode and sequencing adapters with initial denaturation at 95 °C for 10 min, 10 cycles of denaturing at 95 °C for 15 s, annealing at 60 °C for 0.5 min and extension at 68 °C for 1 min, plus a final extension at 68 °C for 3 min.

The concentrations of amplicons were determined using a picogreen assay and a Fluorometer (SpectraMax GeminiXPS 96-well plate reader) and then pooled in equal amounts (~100 ng) into a single tube. The amplicon pool was then cleaned to remove short undesirable fragments using the following procedure. First, the pool was size selected using AMPure beads (Beckman Coulter), the product was then run on a 1 % gel, gel cut and column purified (Qiagen MinElute PCR purification kit), and size selected again with AMPure beads. To determine the final quality, we PCR amplified the resulting amplicon pool with Illumina adaptor-specific primers and ran the PCR product on a DNA 1000 chip for the Agilent 2100 Bioanalyzer. The final amplicon pool was deemed acceptable only if no short fragments were identified after PCR. Otherwise, the procedure was repeated again. The cleaned amplicon pool is then quantified using the KAPA 454 library quantification kit (KAPA Biosciences) and the Applied Biosystems StepOne plus real-time PCR system. Finally, sequences were obtained using an Illumina MiSeq paired-end 300 bp protocol (Illumina, Inc., San Diego, CA, USA) [43].

### Bioinformatics

Raw DNA sequence reads from the Illumina MiSeq were demultiplexed and identified with the custom python application dbcAmplicons (https://github.com/msettles/dbcAmplicons) by both expected barcode and primer sequences. Barcodes were allowed to have at most 1 mismatch (hamming distance) and primers were allowed to have at most 4 mismatches (Levenshtein distance) as long as the final 4 bases of the primer matched the target sequence perfectly. Reads were then trimmed of their primer sequence and merged into a single amplicon sequence using the application flash [44]. Finally, the RDP Bayesian classifier was used to assign sequences to phylotypes [45]. Reads were assigned to the first RDP taxonomic level with a bootstrap score ≥50. The Illumina sequences are available through the National Center for Biotechnology Information (NCBI) Sequence Read Archive (http://www.ncbi.nlm.nih.gov/sra) under project SRP052619.

### Development of kinetic model

Hydrolysis, acidogenesis and methanogenesis were considered for model development in this study. The particulate algal biomass ($S_{\text{p}}$) and dead biomass were hydrolyzed into soluble hydrolysate ($S_{\text{h}}$) by hydrolytic bacteria ($X_{\text{h}}$), then hydrolysate was further degraded into VFA ($S_{\text{v}}$) by acidogenic bacteria ($X_{\text{v}}$); finally, methanogens ($X_{\text{m}}$) convert VFA into methane ($S_{\text{m}}$).

The Contois kinetic model was adopted for all three steps due to improved performance over the first order kinetics [46, 47]. Decay of biomass was considered as first-order kinetics. Non-competitive inhibition type was used as it has been proved to successfully predict inhibition by LCFAs [48, 49]. In this study, the ratio of active biomass plus calcium to LCFAs was adopted by considering that LCFA inhibition is primarily an absorption process onto surface of biomass. According to the above discussion, AD of algal biomass can be described as the following equations:

1 $$\frac{{{\text{d}}S_{\text{p}} }}{{{\text{d}}t}} = - k_{\text{m,p}} \frac{{S_{\text{p}} }}{{K_{\text{s,p}} X_{\text{h}} + S_{\text{p}} }}X_{\text{h}} K_{\text{h}} + k_{\text{d,h}} X_{\text{h}} + k_{\text{d,v}} X_{\text{v}} + k_{\text{d,m}} X_{\text{m}}$$

2 $$\frac{{{\text{d}}S_{\text{h}} }}{{{\text{d}}t}} = k_{\text{m,p}} \frac{{S_{\text{p}} }}{{K_{\text{s,p}} X_{\text{h}} + S_{\text{p}} }}X_{\text{h}} K_{\text{h}} - k_{\text{m,h}} \frac{{S_{\text{h}} }}{{K_{\text{s,h}} X_{\text{v}} + S_{\text{h}} }}X_{\text{v}} K_{\text{v}}$$

3 $$\frac{{{\text{d}}S_{\text{v}} }}{{{\text{d}}t}} = k_{\text{m,h}} \frac{{S_{\text{h}} }}{{K_{\text{s,h}} X_{\text{v}} + S_{\text{h}} }}X_{\text{v}} K_{\text{v}} - k_{\text{m,v}} \frac{{S_{\text{v}} }}{{K_{\text{s,v}} X_{\text{m}} + S_{\text{v}} }}X_{\text{m}} K_{\text{m}}$$

4 $$\frac{{{\text{d}}S_{\text{fa}} }}{{{\text{d}}t}} = f_{\text{fa}} k_{\text{m,p}} \frac{{S_{\text{p}} }}{{K_{\text{s,p}} X_{\text{h}} + S_{\text{p}} }}X_{\text{h}} K_{\text{h}} - k_{\text{m,fa}} \frac{{S_{\text{fa}} }}{{K_{\text{s,fa}} X_{\text{v}} + S_{\text{fa}} }}X_{\text{v}} K_{\text{v}}$$

5 $$\frac{{{\text{d}}S_{\text{m}} }}{{{\text{d}}t}} = k_{\text{m,v}} \frac{{S_{\text{v}} }}{{K_{\text{s,v}} X_{\text{m}} + S_{\text{v}} }}X_{\text{m}} K_{\text{m}}$$

6 $$\frac{{{\text{d}}X_{\text{h}} }}{{{\text{d}}t}} = Y_{\text{h}} k_{\text{m,p}} \frac{{S_{\text{p}} }}{{K_{\text{s,p}} X_{\text{h}} + S_{\text{p}} }}X_{\text{h}} K_{\text{h}} - k_{\text{d,h}} X_{\text{h}}$$

7 $$\frac{{{\text{d}}X_{\text{v}} }}{{{\text{d}}t}} = Y_{\text{v}} k_{\text{m,h}} \frac{{S_{\text{h}} }}{{K_{\text{s,h}} X_{\text{v}} + S_{\text{h}} }}X_{\text{v}} K_{\text{v}} - k_{\text{d,v}} X_{\text{v}}$$

8 $$\frac{{{\text{d}}X_{\text{m}} }}{{{\text{d}}t}} = Y_{\text{m}} k_{\text{m,v}} \frac{{S_{\text{v}} }}{{K_{\text{s,v}} X_{\text{m}} + S_{\text{v}} }}X_{\text{m}} K_{\text{m}} - k_{\text{d,m}} X_{\text{m}}$$

9 $$K_{\text{h}} = \frac{{K_{\text{h,fa}} \left( {\frac{{a(X_{\text{h}} + X_{\text{v}} + X_{\text{m}} ) + bS_{\text{Ca}} }}{{S_{\text{fa}} }}} \right)}}{{K_{\text{h,fa}} \left( {\frac{{a(X_{\text{h}} + X_{\text{v}} + X_{\text{m}} ) + bS_{\text{Ca}} }}{{S_{\text{fa}} }}} \right) + S_{\text{fa}} }}$$

10 $$K_{\text{v}} = \frac{{K_{\text{v,fa}} \left( {\frac{{a(X_{\text{h}} + X_{\text{v}} + X_{\text{m}} ) + bS_{\text{Ca}} }}{{S_{\text{fa}} }}} \right)}}{{K_{\text{v,fa}} \left( {\frac{{a(X_{\text{h}} + X_{\text{v}} + X_{\text{m}} ) + bS_{\text{Ca}} }}{{S_{\text{fa}} }}} \right) + S_{\text{fa}} }}$$

11 $$K_{\text{m}} = \frac{{K_{\text{m,fa}} \left( {\frac{{a(X_{\text{h}} + X_{\text{v}} + X_{\text{m}} ) + bS_{\text{Ca}} }}{{S_{\text{fa}} }}} \right)}}{{K_{\text{m,fa}} \left( {\frac{{a(X_{\text{h}} + X_{\text{v}} + X_{\text{m}} ) + bS_{\text{Ca}} }}{{S_{\text{fa}} }}} \right) + S_{\text{fa}} }}$$

Sensitivity analysis was applied in this study to determine the significance of model parameters and identify the dominant parameters [50]. The relative–relative sensitivity function (*δ*) was used to measure the relative change in methane production for a ± 100% change in kinetic parameters and stoichiometric parameters by Eq. (12) [51].

12 $$\delta = \frac{p/\partial p}{y/\partial y}$$

where $y$ is the given input parameter value, and $p$ is the output of corresponding parameter with a relative change.

The parameters, $k_{\text{m,p}}$, $k_{\text{m,h}}$, $k_{\text{m,fa}}$, $K_{\text{s,p}}$, $K_{\text{s,h}}$, $K_{\text{s, fa}}$, $K_{\text{s,v}}$, $Y_{\text{h}}$, $Y_{\text{v}}$, $k_{\text{d,h}}$ and $k_{\text{d,v}}$ with low sensitivity on model output, were used directly from references without modification in this study [30, 52–54], and their values are presented in Table 2. Other parameters, $k_{\text{m,v}}$, $Y_{\text{m}}$, $k_{\text{d,m}}$, $K_{\text{h, fa}}$, $K_{\text{v, fa}}$, $K_{\text{m, fa}}$, $a$ and $b$ showing significant impact on model output, were estimated according to the batch experimental data.

**Table 2:** Kinetic parameters for anaerobic digestion of NS1 and NS2

Symbol	Units	Initial value
$k_{\text{m,p}}$	day^−1^	10
$k_{\text{m,h}}$	day^−1^	20
$k_{\text{m,fa}}$	day^−1^	6
$k_{\text{m,v}}$	day^−1^	20
$K_{\text{s,p}}$		0.5
$K_{\text{s,h}}$		0.5
$K_{\text{s,fa}}$		0.5
$K_{\text{s,v}}$		0.5
$Y_{\text{h}}$	kgCOD/kgCOD	0.05
$Y_{\text{v}}$	kgCOD/kgCOD	0.05
$Y_{\text{m}}$	kgCOD/kgCOD	0.05
$k_{\text{d,h}}$	day^−1^	0.8
$k_{\text{d,v}}$	day^−1^	0.8
$k_{\text{d,m}}$	day^−1^	0.05
$f_{\text{fa}}$		0.35 (NS1) 0.11 (NS2)
$K_{\text{h, fa}}$	kgCOD/m^3^	5
$K_{\text{v, fa}}$	kgCOD/m^3^	5
$K_{\text{m, fa}}$	kgCOD/m^3^	5
$a$		0.5
$b$		0.5

## Abbreviations

LCFA: long-chain fatty acids
NS1: whole cell algal biomass of *Nannochloropsis**salina*
NS2: lipid-extracted residue of *Nannochloropsis**salina*
AD: anaerobic digestion
*I*/*S*: inoculum to substrate ratio
TS: total solids content
VS: volatile solids content
SMP: specific methane production
COD: chemical oxygen demand
$S_{\text{p}}$: concentration of particulate algal biomass (kg COD/m^3^)
$S_{\text{h}}$: concentration of hydrolysate (kg COD/m^3^)
$S_{\text{v}}$: concentration of VFA (kg COD/m^3^)
$S_{\text{fa}}$: concentration of LCFAs (kg COD/m^3^)
$S_{\text{m}}$: concentration of methane kg COD/m^3^
$S_{\text{Ca}}$: concentration of calcium (kmole/m^3^)
$X_{\text{h}}$: concentration of hydrolytic bacteria (kg COD/m^3^)
$X_{\text{v}}$: concentration of acidogens (kg COD/m^3^)
$X_{\text{m}}$: concentration of methanogens (kg COD/m^3^)
$k_{\text{m,p}}$: maximum specific hydrolysis rate (day^−1^)
$k_{\text{m,h}}$: maximum specific utilization rate of hydrolysate (day^−1^)
$k_{\text{m,fa}}$: maximum specific utilization rate of LCFAs (day^−1^)
$k_{\text{m,v}}$: maximum specific methanogenesis rate (day^−1^)
$K_{\text{s,p}}$: half-saturation coefficient for the ratio $S_{\text{p}} /X_{\text{h}}$
$K_{\text{s,h}}$: half-saturation coefficient for the ratio $S_{\text{h}} /X_{\text{v}}$
$K_{\text{s,fa}}$: half-saturation coefficient for the ratio $S_{\text{fa}} /X_{\text{v}}$
$K_{\text{s,v}}$: half-saturation coefficient for the ratio $S_{\text{v}} /X_{\text{m}}$
$Y_{\text{h}}$: yield coefficient of hydrolytic bacteria (kg COD/kg COD)
$Y_{\text{v}}$: yield coefficient of acidogenic bacteria (kg COD/kg COD)
$Y_{\text{m}}$: yield coefficient of methanogens (kg COD/kg COD)
$k_{\text{d,h}}$: decay rate of hydrolytic bacteria (day^−1^)
$k_{\text{d,v}}$: decay rate of acidogens (day^−1^)
$k_{\text{d,m}}$: decay rate of methanogens (day^−1^)
$f_{\text{fa}}$: stoichiometric coefficient for LCFAs from algal biomass
$K_{\text{h}}$: inhibition factor of LCFAs on hydrolysis step
$K_{\text{v}}$: inhibition factor of LCFAs on acidogenesis step
$K_{m}$: inhibition factor of LCFAs on methanogenesis step
$K_{\text{h, fa}}$: inhibition coefficient of LCFAs on hydrolytic bacteria (kg COD/m^3^)
$K_{\text{v, fa}}$: inhibition coefficient of LCFAs on acidogens (kg COD/m^3^)
$K_{\text{m, fa}}$: inhibition coefficient of LCFAs on methanogens (kg COD/m^3^)
*a*: weight coefficient of absorption of LCFAs by microbes
*b*: weight coefficient of absorption of LCFAs by calcium

## References

[1] Scott SA, Davey MP, Dennis JS, Horst I, Howe CJ, Lea-Smith DJ (2010). Biodiesel from algae: challenges and prospects. Curr Opin Biotech.

[2] Zhao B, Ma J, Zhao Q, Laurens L, Jarvis E, Chen S (2014). Efficient anaerobic digestion of whole microalgae and lipid-extracted microalgae residues for methane energy production. Bioresour Technol.

[3] Sialve B, Bernet N, Bernard O (2009). Anaerobic digestion of microalgae as a necessary step to make microalgal biodiesel sustainable. Biotechnol Adv.

[4] Rinzema A, Boone M, Knippenberg Kv, Lettinga G (1994). Bactericidal effect of long chain fatty acids in anaerobic digestion. Water Environ Res.

[5] Pavlostathis SG, Giraldo-Gomez E (1991). Kinetics of anaerobic treatment: a critical review. Crit Rev Environ Control.

[6] Angelidaki I, Ahring BK (1992). Effects of free long-chain fatty acids on thermophilic anaerobic digestion. Appl Microbiol Biotechnol.

[7] Alves MM, Mota Vieira JA, Álvares Pereira RM, Pereira MA, Mota M (2001). Effects of lipids and oleic acid on biomass development in anaerobic fixed-bed reactors. Part II: oleic acid toxicity and biodegradability. Water Res.

[8] Hanaki K, Matsuo T, Nagase M (1981). Mechanism of inhibition caused by long-chain fatty acids in anaerobic digestion process. Biotechnol Bioeng.

[9] Lalman J, Bagley DM (2002). Effects of C18 long chain fatty acids on glucose, butyrate and hydrogen degradation. Water Res.

[10] Lalman JA, Bagley DM (2000). Anaerobic degradation and inhibitory effects of linoleic acid. Water Res.

[11] Pereira M, Cavaleiro A, Mota M, Alves M (2003). Accumulation of long chain fatty acids onto anaerobic sludge under steady state and shock loading conditions: effect on acetogenic and methanogenic activity. Water Sci Technol.

[12] Nielsen HB, Ahring BK (2006). Responses of the biogas process to pulses of oleate in reactors treating mixtures of cattle and pig manure. Biotechnol Bioeng.

[13] Wu J-T, Chiang Y-R, Huang W-Y, Jane W-N (2006). Cytotoxic effects of free fatty acids on phytoplankton algae and cyanobacteria. Aquat Toxicol.

[14] Zheng CJ, Yoo J-S, Lee T-G, Cho H-Y, Kim Y-H, Kim W-G (2005). Fatty acid synthesis is a target for antibacterial activity of unsaturated fatty acids. FEBS Lett.

[15] Desbois A, Smith V (2010). Antibacterial free fatty acids: activities, mechanisms of action and biotechnological potential. Appl Microbiol Biotechnol.

[16] Templer J, Lalman JA, Jing N, Ndegwa PM (2006). Influence of C18 long chain fatty acids on hydrogen metabolism. Biotechnol Prog.

[17] Pereira MA, Sousa DZ, Mota M, Alves MM (2004). Mineralization of LCFA associated with anaerobic sludge: Kinetics, enhancement of methanogenic activity, and effect of VFA. Biotechnol Bioeng.

[18] Pereira MA, Pires OC, Mota M, Alves MM (2005). Anaerobic biodegradation of oleic and palmitic acids: evidence of mass transfer limitations caused by long chain fatty acid accumulation onto the anaerobic sludge. Biotechnol Bioeng.

[19] Zonta Ž, Alves MM, Flotats X, Palatsi J (2013). Modelling inhibitory effects of long chain fatty acids in the anaerobic digestion process. Water Res.

[20] Fernández A, Sanchez A, Font X (2005). Anaerobic co-digestion of a simulated organic fraction of municipal solid wastes and fats of animal and vegetable origin. Biochem Eng J.

[21] Palatsi J, Laureni M, Andrés M, Flotats X, Nielsen H, Angelidaki I (2009). Strategies for recovering inhibition caused by long chain fatty acids on anaerobic thermophilic biogas reactors. Bioresour Technol.

[22] Cavaleiro A, Pereira M, Alves M (2008). Enhancement of methane production from long chain fatty acid based effluents. Bioresour Technol.

[23] Roy F, Albagnac G, Samain E (1985). Influence of calcium addition on growth of highly purified syntrophic cultures degrading long-chain fatty acids. Appl Environ Microbiol.

[24] Hatamoto M, Imachi H, Ohashi A, Harada H (2007). Identification and cultivation of anaerobic, syntrophic long-chain fatty acid-degrading microbes from mesophilic and thermophilic methanogenic sludges. Appl Environ Microbiol.

[25] Ahn J-H, Do TH, Kim SD, Hwang S (2006). The effect of calcium on the anaerobic digestion treating swine wastewater. Biochem Eng J.

[26] Koster IW, Cramer A (1987). Inhibition of methanogenesis from acetate in granular sludge by long-chain fatty acids. Appl Environ Microbiol.

[27] Neves L, Oliveira R, Alves MM (2009). Fate of LCFA in the co-digestion of cow manure, food waste and discontinuous addition of oil. Water Res.

[28] Astals S, Batstone DJ, Mata-Alvarez J, Jensen PD (2014). Identification of synergistic impacts during anaerobic co-digestion of organic wastes. Bioresour Technol.

[29] Palatsi J, Illa J, Prenafeta-Boldú FX, Laureni M, Fernandez B, Angelidaki I (2010). Long-chain fatty acids inhibition and adaptation process in anaerobic thermophilic digestion: batch tests, microbial community structure and mathematical modelling. Bioresour Technol.

[30] Angelidaki I, Ellegaard L, Ahring BK (1999). A comprehensive model of anaerobic bioconversion of complex substrates to biogas. Biotechnol Bioeng.

[31] Wakelin NG, Forster CF (1997). An investigation into microbial removal of fats, oils and greases. Bioresour Technol.

[32] Mongkolthanaruk W, Dharmsthiti S (2002). Biodegradation of lipid-rich wastewater by a mixed bacterial consortium. Int Biodeterior Biodegrad.

[33] Assih EA, Ouattara AS, Thierry S, Cayol J-L, Labat M, Macarie H (2002). Stenotrophomonas acidaminiphila sp. nov., a strictly aerobic bacterium isolated from an upflow anaerobic sludge blanket (UASB) reactor. Int J Syst Evol Microbiol.

[34] Zamalloa C, De Vrieze J, Boon N, Verstraete W (2012). Anaerobic digestibility of marine microalgae Phaeodactylum tricornutum in a lab-scale anaerobic membrane bioreactor. Appl Microbiol Biotechnol.

[35] Labatut R, Angenent L, Scott N (2011). Biochemical methane potential and biodegradability of complex organic substrates. Bioresour Technol.

[36] APHA (1998). Methods for examination of water and wasterwater.

[37] Ma J, Yu L, Frear C, Zhao Q, Li X, Chen S (2013). Kinetics of psychrophilic anaerobic sequencing batch reactor treating flushed dairy manure. Bioresour Technol.

[38] Laurens LL, Quinn M, Wychen S, Templeton D, Wolfrum E (2012). Accurate and reliable quantification of total microalgal fuel potential as fatty acid methyl esters by in situ transesterification. Anal Bioanal Chem.

[39] Lourenço SO, Barbarino E, Lavín PL, Lanfer Marquez UM, Aidar E (2004). Distribution of intracellular nitrogen in marine microalgae: calculation of new nitrogen-to-protein conversion factors. Eur J Phycol.

[40] Laurens LM, Dempster TA, Jones HD, Wolfrum EJ, Van Wychen S, McAllister JS (2012). Algal biomass constituent analysis: method uncertainties and investigation of the underlying measuring chemistries. Anal Chem.

[41] Huber JA, Mark Welch DB, Morrison HG, Huse SM, Neal PR, Butterfield DA (2007). Microbial population structures in the deep marine biosphere. Science.

[42] Marteinsson VT, Runarsson A, Stefansson A, Thorsteinsson T, Johannesson T, Magnusson SH et al (2013) Microbial communities in the subglacial waters of the Vatnajokull ice cap, Iceland. ISME J. 7(2):427–437. http://www.nature.com/ismej/journal/v7/n2/suppinfo/ismej201297s1.html 10.1038/ismej.2012.97 PMC3554413 22975882

[43] Caporaso JG, Lauber CL, Walters WA, Berg-Lyons D, Huntley J, Fierer N et al (2012) Ultra-high-throughput microbial community analysis on the Illumina HiSeq and MiSeq platforms. ISME J 6(8):1621–1624. http://www.nature.com/ismej/journal/v6/n8/suppinfo/ismej20128s1.html 10.1038/ismej.2012.8 PMC3400413 22402401

[44] Magoč T, Salzberg SL (2011). FLASH: fast length adjustment of short reads to improve genome assemblies. Bioinformatics.

[45] Wang Q, Garrity GM, Tiedje JM, Cole JR (2007). Naïve Bayesian classifier for rapid assignment of rRNA sequences into the new bacterial taxonomy. Appl Environ Microbiol.

[46] Vavilin VA, Fernandez B, Palatsi J, Flotats X (2008). Hydrolysis kinetics in anaerobic degradation of particulate organic material: an overview. Waste Manag.

[47] Ramirez I, Mottet A, Carrère H, Déléris S, Vedrenne F, Steyer J-P (2009). Modified ADM1 disintegration/hydrolysis structures for modeling batch thermophilic anaerobic digestion of thermally pretreated waste activated sludge. Water Res.

[48] Gavala H, Angelidaki I, Ahring B. Kinetics and Modeling of Anaerobic Digestion Process. In: Ahring B, Angelidaki I, Macario EC, Gavala HN, Hofman-Bang J, Macario AJL et al (2003) Biomethanation I. Advances in Biochemical Engineering/Biotechnology. Springer Berlin Heidelberg, pp 57–93 10.1007/3-540-45839-5_3 12747561

[49] Zhang P, Chen Y, Zhou Q, Zheng X, Zhu X, Zhao Y (2010). Understanding short-chain fatty acids accumulation enhanced in waste activated sludge alkaline fermentation: kinetics and microbiology. Environ Sci Technol.

[50] Tartakovsky B, Mu SJ, Zeng Y, Lou SJ, Guiot SR, Wu P (2008). Anaerobic digestion model No. 1-based distributed parameter model of an anaerobic reactor: II. Model validation. Bioresour Technol.

[51] Reichert P (1998) AQUASIM 2.0: Computer program for the identification and simulation of aquatic systems. Swiss Federal Institute for Environmental Science and Technology (EAWAG) Dubendorf, Switzerland

[52] Blumensaat F, Keller J (2005). Modelling of two-stage anaerobic digestion using the IWA Anaerobic Digestion Model No. 1 (ADM1). Water Res.

[53] Batstone DJ, Keller J, Angelidaki I, Kalyuzhnyi SV, Pavlostathis SG, Rozzi A et al (2002) Anaerobic digestion model no.1. Scientific and Technical Report 9. International Water Association (IWA), London 12188579

[54] Siegrist H, Vogt D, Garcia-Heras JL, Gujer W (2002). Mathematical Model for Meso- and Thermophilic Anaerobic Sewage Sludge Digestion. Environ Sci Technol.

